# Pimavanserin: A Novel Autophagy Modulator for Pancreatic Cancer Treatment

**DOI:** 10.3390/cancers13225661

**Published:** 2021-11-12

**Authors:** Sharavan Ramachandran, Itishree S. Kaushik, Sanjay K. Srivastava

**Affiliations:** Department of Immunotherapeutics and Biotechnology, Center for Tumor Immunology and Targeted Cancer Therapy, Texas Tech University Health Sciences Center, Abilene, TX 79601, USA; sharavan.ramachandran@ttuhsc.edu (S.R.); i.kaushik@ttuhsc.edu (I.S.K.)

**Keywords:** cancer, autophagy, cell death, drug repurposing, apoptosis

## Abstract

**Simple Summary:**

Pimavanserin a novel anti-psychotic drug suppresses the growth of pancreatic tumors in vitro and in vivo and can be developed as a treatment option for pancreatic cancer.

**Abstract:**

Pancreatic tumors exhibit high basal autophagy compared to that of other cancers. Several studies including those from our laboratory reported that enhanced autophagy leads to apoptosis in cancer cells. In this study, we evaluated the autophagy and apoptosis inducing effects of Pimavanserin tartrate (PVT). Autophagic effects of PVT were determined by Acridine Orange assay and Transmission Electron Microscopy analysis. Clinical significance of ULK1 in normal and pancreatic cancer patients was evaluated by R2 and GEPIA cancer genomic databases. Modulation of proteins in autophagy signaling was assessed by Western blotting and Immunofluorescence. Apoptotic effects of PVT was evaluated by Annexin-V/APC assay. Subcutaneous xenograft pancreatic tumor model was used to evaluate the autophagy-mediated apoptotic effects of PVT in vivo. Autophagy was induced upon PVT treatment in pancreatic ducal adenocarcinoma (PDAC) cells. Pancreatic cancer patients exhibit reduced levels of autophagy initiator gene, ULK1, which correlated with reduced patient survival. Interestingly, PVT induced the expression of autophagy markers ULK1, FIP200, Atg101, Beclin-1, Atg5, LC3A/B, and cleavage of caspase-3, an indicator of apoptosis in several PDAC cells. ULK1 agonist LYN-1604 enhanced the autophagic and apoptotic effects of PVT. On the other hand, autophagy inhibitors chloroquine and bafilomycin blocked the autophagic and apoptotic effects of PVT in PDAC cells. Notably, chloroquine abrogated the growth suppressive effects of PVT by 25% in BxPC3 tumor xenografts in nude mice. Collectively, our results indicate that PVT mediated pancreatic tumor growth suppression was associated with induction of autophagy mediated apoptosis.

## 1. Introduction

Several stress elements including drug-induced stress activate intra-cellular survival mechanisms such as autophagy to tolerate the harsh conditions or trigger cell death pathways [1,2]. Autophagy occurs in three forms such as; macroautophagy, microautophagy, and chaperone-mediated autophagy. Macroautophagy, hereafter referred to as autophagy, is the most prevalent form of autophagy in eukaryotes, and play a pivotal role in maintaining tissue homeostasis. Autophagy is considered as an evolutionarily conserved catabolic process, where the sequestered cellular organelles are delivered to lysosomes by the autophagosomes for degradation. This ubiquitous process happens in five distinct stages: initiation, vesicle nucleation, vesicle elongation, autophagosome expansion, and fusion of autophagosome with lysosome for degradation. ULK1 orchestrates the initiation of autophagy, where it assembles with Atg13, Atg101, and FIP200 to form the autophagy initiation complex. Phosphorylation of ULK1 at Ser 757 prevents ULK1 in forming the autophagy initiation complex, thereby inhibits autophagy. The ULK1 complex guides the assembly of class-III PI3K or VPS34 complex comprising VPS34, Atg14, UVRAG and AMBRA1 scaffolded by tumor suppressor, Beclin-1. This kinase complex is responsible for the formation of double membraned structures called autophagosomes and mediates membrane nucleation. Subsequently, Atg4, Atg7, and Atg3 proteins facilitate the formation of Atg5-Atg12-Atg16 complex. This assembly is recruited to the precursor autophagosomal structure to prevent premature association of autophagosomes with lysosomes. In a ubiquitin-like conjugation system, Atg5-Atg12-Atg16 complex conjugates phosphatidylethanolamine (PE) with Atg8/microtubule-associated protein 1 light chain 3 (LC3). This lipidation of LC3 leads to the formation of LC3-II, which is incorporated in the autophagosome membrane, thereby promoting the elongation and closure of autophagosome membrane. LC3-II serves as an autophagosomal marker. Eventually, the autophagosome fuses with the acidified lysosomes to form autophagolysosomes, where the sequestered cellular cargo and organelles are degraded by acidic hydrolases [3,4,5,6].

Autophagy dysfunction is considered to be one of the hallmarks of PDAC progression. PDAC possesses a high level of basal autophagy. Recent studies reported autophagy as a pro-survival mechanism in cellular defense and tumor metabolism to fuel pancreatic tumor growth. Nonetheless, inducing autophagy beyond threshold can lead to apoptotic cell death [7,8,9,10]. Given the contradictory roles of autophagy in cancer, the connection between autophagy and apoptosis is complex and warrants further studies.

Clinical interventions at various stages of autophagy were proposed as a potential therapeutic strategy for cancer therapy. Gemcitabine (first line treatment for PDAC) was shown to inhibit pancreatic tumor growth by inducing autophagy-mediated apoptotic cell death [9]. However, PDAC patients acquire resistance to gemcitabine and it is considered as one of major obstacles in the treatment of PDAC. Antipsychotic drugs were shown to induce neuronal autophagy in several neurological disorders contributing to the improvement in neuronal functions. Antipsychotic drugs like penfluridol, reserpine, thioridazine, fluspirilene, chlorpromazine, pimozide, trifluoperazine, fluphenazine, methotrimeprazine, olanzapine, sertindole, clozapine, and haloperidol were shown to induce autophagy in normal neuronal and cancer cells [10,11,12].

In our previous study, we demonstrated that a novel anti-Parkinson/psychotic drug, Pimavanserin tartrate (PVT) suppressed the growth of pancreatic tumors by inducing apoptosis in vitro and in vivo through suppression of Akt/Gli-1 signaling axis [13]. Several reports demonstrated the role of Akt in autophagy and apoptosis [14,15,16]. In the current study, we observed activation of ULK1-mediated autophagy as a mechanism of apoptotic effects of PVT in pancreatic cancer. Oral administration of PVT significantly suppressed the growth of pancreatic tumor xenografts by inducing ULK1-mediated autophagy. The autophagy inhibitor chloroquine abrogated the tumor growth suppressive effects of PVT by rescuing PDAC cells from PVT-induced apoptosis. To the best of our knowledge, this study is the first to unveil the proautophagic role of PVT in pancreatic cancer.

## 2. Materials and Methods

### 2.1. Ethics Statement

All the preclinical investigations involving animals were carried out in line with the ethical standards and according to the approved protocol by Institutional Animal Care and Use Committee (IACUC). The animal studies were conducted in Texas Tech University Health Sciences Center LARC facility, Abilene.

### 2.2. Cell Culture

Human PDAC cell lines AsPC1, BxPC3, and MIAPaCa2 and murine pancreatic cancer cell line PO2 were procured and cultured as mentioned in our previous study [13]. Monolayer cultures of L3.6pl cells were cultured in MEM supplemented with 10% fetal bovine serum, 1% penicillin-streptomycin antibiotic mixture, 2 mM L-glutamine, 10 mM HEPES, 1 mM sodium pyruvate, 2.2 g/L sodium bicarbonate, and 10 mL/L glucose. All the cells were cultured at 37 °C incubator with 5% CO_2_.

### 2.3. Cytotoxicity Assay

Cytotoxic effects of PVT was evaluated in L36.pl cells by SRB staining. The cells were seeded, treated, and processed for SRB assay as described by us previously [13].

### 2.4. Colony Formation Assay

PO2 pancreatic cancer cells were seeded at a density of 500 cells per well in a 6-well plate and incubated overnight. Cells were then treated with increasing concentrations of PVT (0–7.5 µM) for 24 h. After 24 h, cells were replenished with 2 mL fresh medium and incubated at 37 °C, 5% CO_2_ for 2 weeks. Cells were then processed by TCA fixation and SRB staining as described in our previous study [13]. The colonies (*n* = 3) were quantified using Image J software.

### 2.5. Acridine Orange Assay Using Flow Cytometry

Briefly, 0.1–0.2 × 10^6^ cells per well were seeded in a 6-well plate and incubated overnight for cell attachment. Next day, cells were subjected to PVT treatment (0–7.5 μM) for 24 h. Cells were then trypsinized using 0.25% trypsin and washed twice using 1× PBS. The cell pellet was suspended in 500 μL of 1× PBS and then incubated with 1.5 μM acridine orange in dark for 30 min. Readings were recorded using flow cytometry (BD LSR Fortessa, BD biosciences, San Jose, CA, USA). Flow cytometry data were analyzed using Flowjo software and the data were plotted using Graphpad Prism. All the experiments were repeated independently for 2–3 times.

### 2.6. Acridine Orange Assay Using Confocal Microscopy

AsPC1 cells were seeded in a poly-L-lysine-coated cover slip and treated under similar conditions as stated in Section 2.5. The cover slips containing cells were removed and placed in a fresh 6-well plate and washed twice with 1× PBS for 5 min. Cells were then incubated with 1.5 μM acridine orange in dark for 30 min. The cover slips were then mounted on a glass slide containing one drop of NucBlue live cell stain (Invitrogen). Cover slips were sealed to the glass slide using an adhesive and was allowed to dry at room temperature in dark for 10 min. The fixed cover slips were then analyzed for acridine orange staining using confocal microscopy (Nikon confocal microscope A1, Nikon, Melville, NY, USA).

### 2.7. LysoTracker Assay Using Confocal Microscopy

Cells were seeded and treated under similar conditions as stated in Section 2.6 Cells were stained with 50 nM LysoTracker Green DND-26 dye for 15 min. Following staining step, cover slips were fixed with 4% paraformaldehyde and washed twice with 1× PBS. The coverslips were then mounted on a glass slide using DAPI mounting medium and dried at room temperature. The images were taken using a confocal microscope (Nikon confocal microscope A1) at 100× magnification.

### 2.8. Electron Microscopy

0.7 × 10^6^ AsPC1 cells were seeded in a petri-dish and allowed for overnight attachment. Cells were treated with 7.5 µM PVT for 24 h. Cells were then trypsinized and washed twice with ice-cold 1× PBS. Cell pellet was resuspended in 1:1 fixative agent (2% Paraformaldehyde (PFA)/2.5% Glutaraldehyde in 0.1 M Sodium Cacodylate Buffer, pH 7.4): 1× PBS i.e., 100 µL fixative agent: 100 µL 1× PBS. Resuspended pellet was then stored in 4 °C and sent to Texas Tech University Health Sciences Center, Lubbock for analysis.

### 2.9. Cancer Genomic Data Analysis

Basal expression of ULK1 in clinical samples was evaluated using R2 and GEPIA public genomic databases [17,18]. In R2 database, 504 normal tissues and 32 pancreatic tumor tissues were considered for genomic analysis of ULK1. R2 data were transformed into rank for further analysis. In GEPIA database, pancreatic adenocarcinoma (PAAD) dataset was selected for analysis. The dataset comprised of 171 normal pancreas and 179 pancreatic tumor tissues. The parameters for expression analysis were as follows, Log_2_FC cutoff: 1; *p*-value cutoff: 0.01. The role of ULK1 in overall patient survival was determined by GEPIA database. This analysis was performed in PAAD dataset which comprised of 89 patients in each group (low ULK1, high ULK1). Samples with expression level higher than 50% were taken as high-expression cohort (Cutoff-High (%): 50), whereas samples with expression level lower than 50% were considered as low-expression cohort (Cutoff-Low (%): 50). Hazards ratio was calculated using Cox PH model [18].

### 2.10. Western Blotting

Western blotting was performed in whole cell lysates of AsPC1, BxPC3, L3.6pl, MIAPaCa2, and PO2 cells treated with 2.5, 5, and 7.5 µM PVT for 24 h SDS-PAGE resolved proteins were immunoblotted for ULK1, pULK1(S757), Atg101, FIP200, Beclin-1, Atg-5, LC3A/B, cleaved caspase-3 and β-actin (Cell Signaling Technologies, Danvers, MA, USA). All experiments were repeated for at least 2 times.

### 2.11. Immunofluorescence

Approximately, 0.2 million cells were seeded on a poly-l-lysine coated coverslip in a 6-well plate. The cells were incubated at 37 °C and 5% CO_2_ for 24 h, after which the cells were treated with 7.5 µM PVT. After 24 h of PVT treatment, the coverslips were placed in a fresh 6 well plate containing 1 mL of 1× PBS. The cells were then given 3 washes with fresh 1× PBS for 5 min each. After washing, the cells were fixed with 4% PFA for 10 min at room temperature. After incubation with PFA, cells were again subjected to three 1× PBS washes for 5 min each. The cells were then permeabilized using 0.1% Triton ×100 for 10 min and washed thrice with 1× PBS (5 min each). After the permeabilization step, the cells were then blocked with 1% goat serum, 0.25% Tween 20 in PBS for 1 h. Cells were then incubated with ULK1 and LC3B antibodies in blocking buffer for overnight at 4 °C in a hydrated chamber. Next day, cells were washed four times using 1× PBS. The cells were then incubated in secondary antibody Alexa Fluor 488 (Cat No.: ab150077) diluted at a ratio of 1:1000 in 3% goat serum and 0.5% BSA. After secondary antibody incubation, cells were washed with 1× PBS for 5 min. After washing, cells were incubated in Alexa Fluor 594 phalloidin (Cat No.: A12381) diluted at a ratio of 1:500 in 3% BSA for 15 min. Once the phalloidin incubation was completed, the coverslips were gently tapped on a tissue paper and mounted on a glass slide containing one drop of ProLong Diamond Antifade Mountant with DAPI (Cat No.: P36941). The slides were left at room temperature for 2 h and then stored at 4 °C. Images were taken using confocal microscope (Nikon confocal microscope A1).

### 2.12. Annexin-V/APC Assay

Apoptosis assay was performed by AnnexinV/APC apoptosis detection kit (BD biosciences, San Jose, CA, USA). 0.1–0.2 × 10^6^ PDAC cells were treated with increasing concentrations of PVT (0–7.5 μM) for 24 h. At the desired time points, cells were trypsinized and then analyzed for Annexin and PI staining using flow cytometry (BD LSR Fortessa, BD biosciences, San Jose, CA, USA). The flow cytometry data were integrated into the FlowJo software and % apoptotic cells were quantified. All the experiments were repeated independently for 2–3 times.

### 2.13. LC3B Silencing, LYN-1604 and Bafilomycin Treatment

AsPC1, BxPC3 or PO2 cells were seeded at a density of 0.2–0.7 × 10^6^ cells in a 6-well plate or petri dish. After overnight attachment, cells were pretreated with 10µM LYN-1604 or 10 nM Bafilomycin for 3 h. LC3B silencing was achieved in BxPC3 cells by transfecting LC3B siRNA (Cell Signaling Technologies, Danvers, MA, USA) using siPORT transfection reagent according to manufacturer’s instructions. Cells were then treated with 7.5 µM PVT for 24 h and analyzed by western blotting or acridine orange staining.

### 2.14. Subcutaneous Implantation of Pancreatic Tumor Xenografts

Briefly, 1 million BxPC3 cells were implanted subcutaneously in a 6-week old female athymic nude mice. Once the tumors reached to 70 mm^3^ in size, mice were randomized into 4 groups, control (group 1), Chloroquine (group 2), PVT (group 3) and Chloroquine + PVT (group 4). Mice were pre-treated with 25 mg/kg Chloroquine (i.p) for 3 days (group 2 & 4) and then treated with 10 mg/kg PVT by oral gavage daily until day 33 (group 3 & 4). 28 female athymic nude mice were used in this study with each group containing 6 mice. All the authors were aware of the group allocation in all stages of the experiment. Tumor volume was measured periodically using a Vernier caliper. Mice were sacrificed at day 33 due to tumor burden. Aseptically excised tumors were weighed and snap frozen for western blotting analysis.

### 2.15. Immunohistochemistry (IHC)

The formalin fixed paraffin embedded tissues were sectioned into 5 µm slices using a microtome. The slides were processed as described by us previously [13]. The data were quantitated using scan scope and plotted using GraphPad Prism software.

### 2.16. Statistical Analysis

Statistical analysis was performed by Prism 7.0 software (GraphPad software Inc., San Diego, CA, USA). Results are represented as mean ± standard deviation (SD) for in vitro experiments or standard error mean (SEM) for in vivo experiments. Statistical significance was analyzed using Student’s *t*-test followed by two-way ANOVA and the outcomes were considered statistically significant at *p* ≤ 0.05. Statistical significance for R2 database was evaluated by Kruskal–Wallis Test. Sample size for the in vivo experiment was calculated using power and sample size calculation software developed by DuPont and Plummer [19].

## 3. Results

### 3.1. Induction of Autophagy by PVT

**Figure 1:**
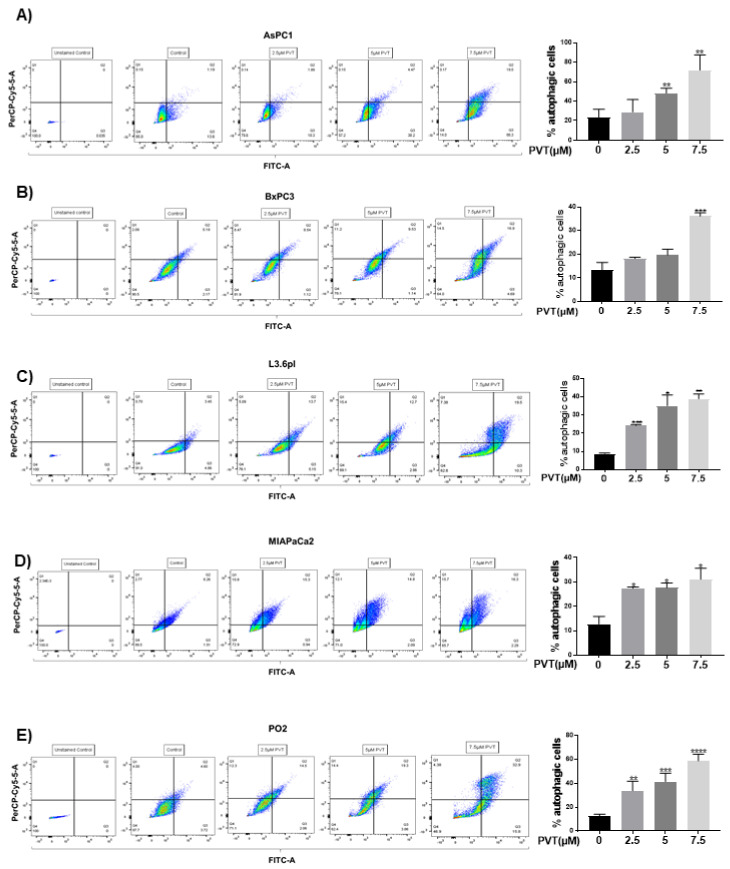
PVT treatment increases autophagy in PDAC cells. Autophagic effects of PVT in (**A**) AsPC1, (**B**) BxPC3, (**C**) L3.6pl, (**D**) MIAPaCa2, and (**E**) PO2 cells were evaluated by acridine orange assay using flow cytometry. All experiments were independently repeated for 3 times. Data were quantitated using FlowJo software and plotted using GraphPad Prism 7.0. * *p* ≤ 0.05, ** *p* ≤ 0.01, *** *p* ≤ 0.001, **** *p* ≤ 0.0001.

**Figure 2:**
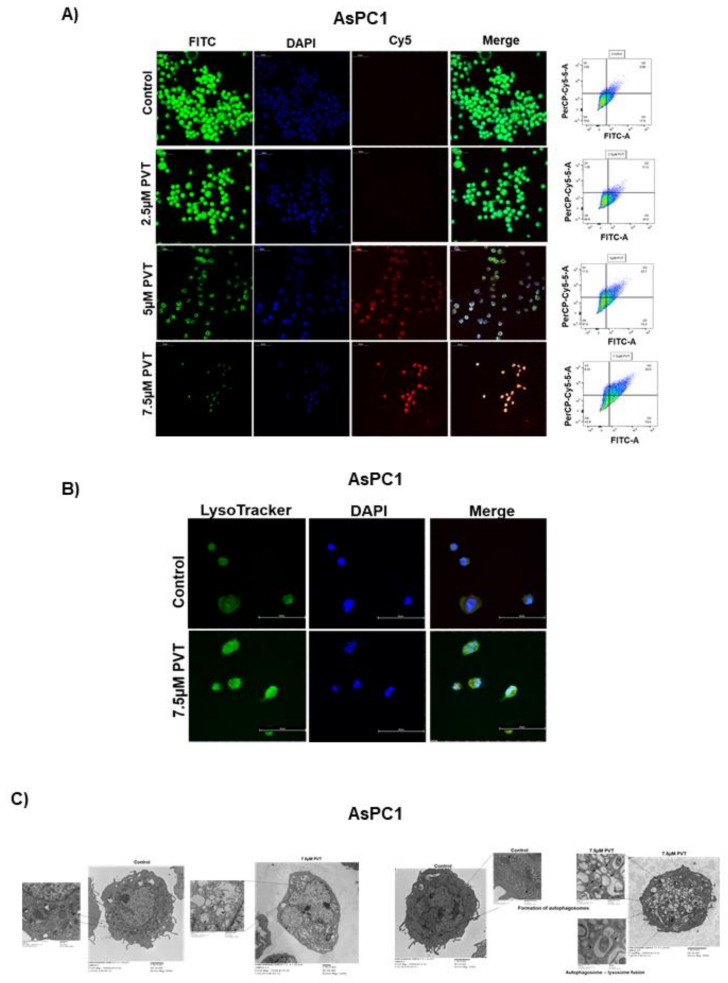
PVT treatment induces autophagosome formation, lysosomal acidification, and fusion of autophagosome with lysosome. (**A**) Autophagy induction by PVT treatment was confirmed by confocal microscopy in AsPC1 cells. (**B**) Localization of acidic lysosomes was confirmed by LysoTracker assay in AsPC1 cells treated with 7.5 µM PVT. (**C**) Electron microscopic images of control and PVT treated AsPC1 cells showing effect of PVT on autophagosome formation and autophagosome—lysosome fusion. Statistically significant when compared with that of control.

Initially, we determined the effects of PVT in modulating autophagy by acridine orange (AO) assay. AO is a cell permeable green fluorophore, which becomes protonated and trapped in acidic vesicular organelles (AVOs). This entrapment of AO in AVO causes a metachromatic shift of AO from green to red fluorescence in a concentration dependent manner [20]. AO staining is a rapid and reliable method to determine the volume of AVOs. The metachromatic shift to red fluorescence denotes AVOs like autolysosomes, which is elevated upon autophagy induction [20,21]. Treatment of AsPC1, BxPC3, L3.6pl, MIAPaCa2 and PO2 cells with increasing concentrations of PVT resulted in a significant increase in the proportion of autophagic cells (Figure 1A–E). For instance, treatment with 7.5 µM PVT resulted in a 3.0 fold increase in AO staining in AsPC1 and BxPC3 cells (Figure 1A,B), whereas a 2.5–5.0 fold autophagy induction was observed in L3.6pl, MIAPaCa2 and PO2 cells (Figure 1C–E). Similarly, 5 μM PVT caused 1.5–2.0 fold increase in autophagy in AsPC1, BxPC3 and MIAPaCa2 cells (Figure 1A,B,D). Same concentrations of PVT induced 3.4 and 4.0 fold autophagy in PO2 and L3.6pl cells respectively (Figure 1C,E). The pro-autophagic effects of PVT were confirmed by confocal microscopy. Our results showed that PVT displayed a concentration-dependent increase in the staining of AO (Cy5) in AsPC1 cells. Cells from the same experiment were also analyzed for AO staining using flow cytometry. Our results showed an increase in AO staining with PVT treatment confirming our microscopy results (Figure 2A). In addition, the effect of PVT on the acidification of lysosomes was confirmed by LysoTracker assay using LysoTracker Green DND-26 dye. The dye accumulates in the lysosomes and the intensity of green staining directly correlates with the acidification of lysosomes. Treatment of AsPC1 cells with 7.5 µM PVT significantly increased the acidification of lysosomes as shown by enhanced green staining (Figure 2B). Moreover, treatment with 7.5 µM PVT increased 30% of LC3 puncta in PDAC cells (Figure 2A). Autophagy induction by PVT was further validated by transmission electron microscopy in AsPC1 cells. As outlined in Figure 2C PVT induced the formation of autophagosomes as represented by the accumulation of double membrane vesicles, shown by black arrows. Furthermore, PVT induced the fusion of autophagosome with lysosome, which clearly demonstrates the role of PVT in inducing late stage autophagy (Figure 2C).

### 3.2. ULK1 Is Downregulated in Pancreatic Tumor Tissues

**Figure 3:**
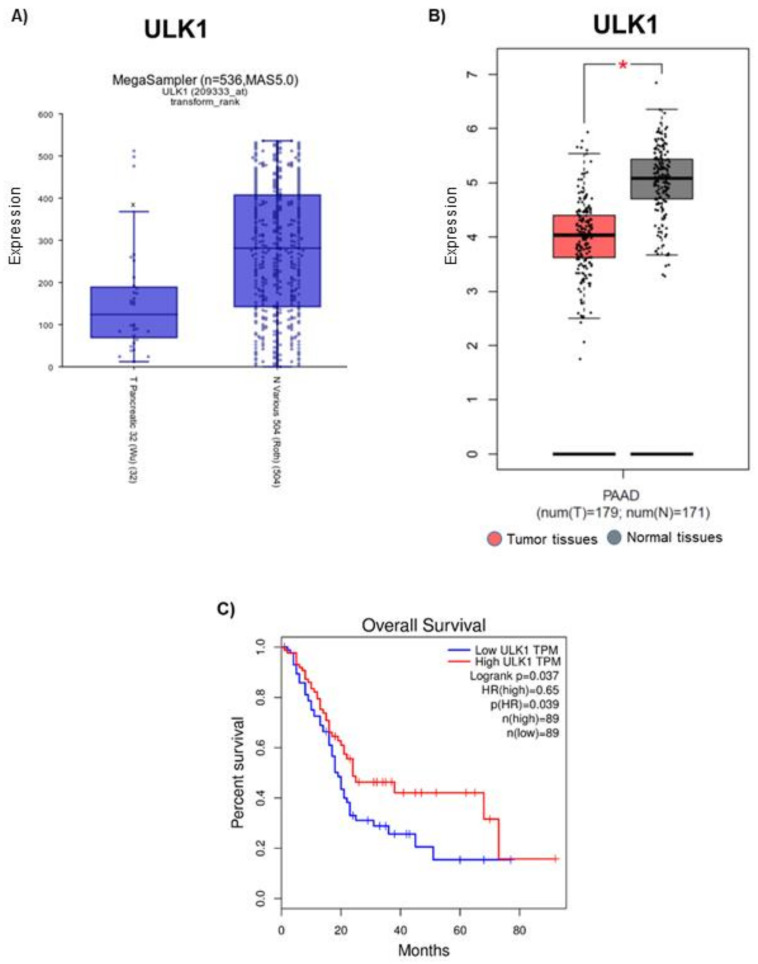
Expression of ULK1 in PDAC patient samples and its effect on overall survival rate. Basal expression of ULK1 in pancreatic tumor patients was evaluated by Cancer Genomic databases (**A**) R2 and (**B**) GEPIA. Data are represented as fold change with respect to healthy tissues. Role of ULK1 in (**C**) Overall patient survival was assessed using GEPIA database. * *p* ≤ 0.05.

Analysis of Cancer genomic R2 and GEPIA databases revealed that ULK1 was differentially expressed in human pancreatic tumors when compared to that of normal tissues. R2 software analysis demonstrated that pancreatic cancer patients exhibited 56% reduced expression of ULK1, when compared with normal patients (Figure 3A). Similarly, GEPIA database suggests that ULK1 was significantly downregulated in human pancreatic tumor tissues compared to that of normal tissues (Figure 3B). Overall patient survival serves as a crucial factor in determining the prognosis of differentially expressed genes. GEPIA database revealed that patients with high ULK1 expression had better survival rate than patients with low ULK1 expression. The 5-year survival rate was approximately 40% in pancreatic cancer patients with high ULK1 expression as compared to <20% in low ULK1 expressing pancreatic cancer patients (Figure 3C). The 0.65 hazard ratio from this study indicated that ULK1 expression reduces the risk of death by 35% (Figure 3C). These findings demonstrate the significance of ULK1 in the clinical setting.

### 3.3. PVT Induces ULK1-Mediated Autophagy in PDAC Cells

**Figure 4:**
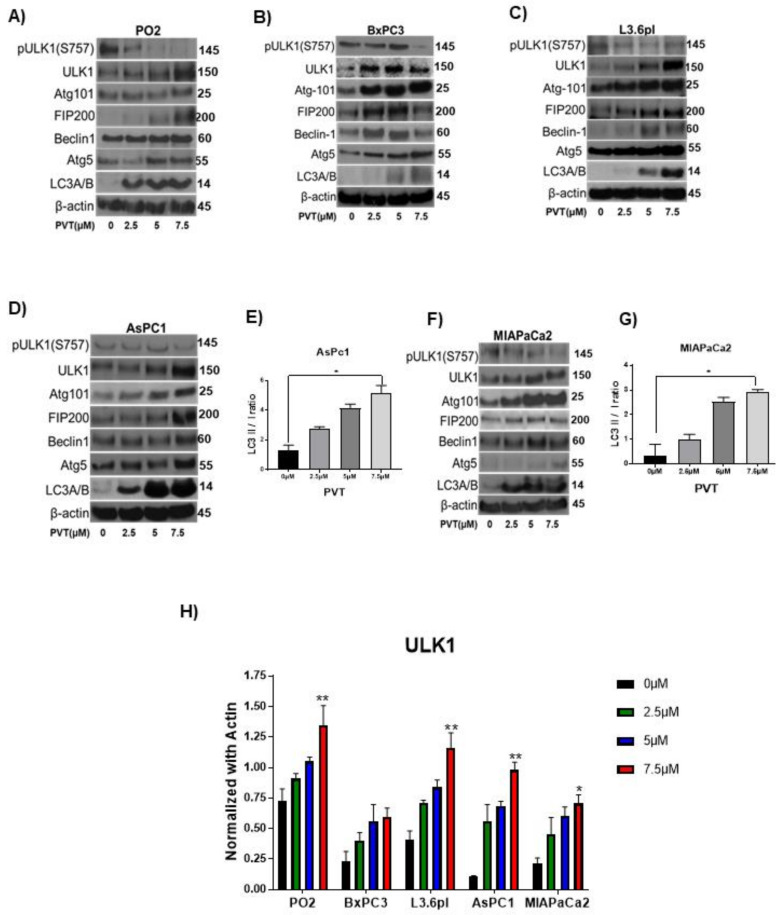
PVT elevates ULK1 mediated autophagy in PDAC cells. Western blot analysis of pULK1(S757), ULK1, Atg101, FIP200, Beclin-1, Atg5, and LC3A/B in (**A**) PO2 (**B**) BxPC3 (**C**) L3.6pl, (**D**) AsPC1, and (**F**) MIAPaCa2 cells treated with 2.5, 5 and 7.5 µM PVT for 24 h. β-actin was used as a loading control. Figures shown are representative blots of three independent experiments. Blots were developed using Optimax X-ray film processor (Protec, Germany). PVT enhanced LC3 II/I ratio in (**E**) AsPC1 and (**G**) MIAPaCa2 cells treated with PVT for 24 h. Proteins were probed for LC3 antibodies and LC3 II/I band density was measured using Image J. (**H**) Quantitated representation of ULK1 expression data obtained from Figure 4A–D,F. * *p* ≤ 0.05, ** *p* ≤ 0.01.

**Figure 5:**
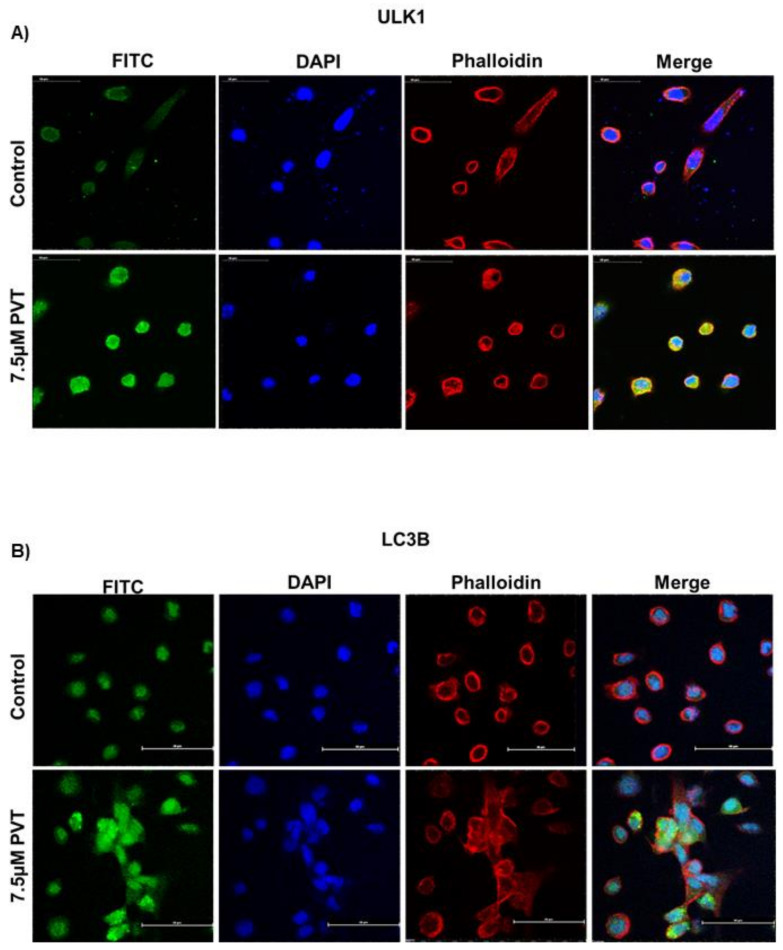
Confocal microscopy confirms autophagy induction in PVT treated PDAC cells. Immunofluorescence analysis of (**A**) ULK1 and (**B**) LC3B (FITC—green), Nucleus (DAPI—blue), Phalloidin (TRITC—Red) in AsPC1 cells treated with 7.5 µM PVT for 24 h.

To gain further insight into the molecular mechanism of autophagic effects of PVT, we performed western blot analysis in whole cell lysates of AsPC1, BxPC3, L3.6pl, MIAPaCa2, and PO2 cells treated with 0, 2.5, 5, and 7.5 μM PVT for 24 h. Results from this experiment showed that, PVT enhanced the expression of autophagy initiation complex markers ULK1, FIP200, Atg101; class-III PI3K complex markers Beclin-1 and autophagosome formation markers Atg-5 and LC3A/B in all the cell lines tested. In addition, PVT suppressed the phosphorylation of ULK1 at Ser 757, which allowed ULK1 to form the initiation complex and initiate autophagy as exhibited by an increase in ULK1 expression with PVT treatment (Figure 4A–F). To further validate the increased production of autophagosome upon PVT treatment, the conversion of LC3 I to LC3 II was measured by quantitating the band density of immunoblots. Our results indicate that treatment with PVT increased the LC3 II/I ratio in PDAC cells in a concentration dependent manner (Figure 4E,G). Modulation of ULK1 and LC3B was further confirmed by immunofluorescence, showing that 7.5 μM PVT elevated the expression of ULK1 and LC3B, as depicted by increased green staining when compared with control (Figure 5A,B). These results confirmed the induction of autophagy by PVT.

### 3.4. ULK1 Agonist LYN-1604 Potentiates the Autophagic Effects of PVT

**Figure 6:**
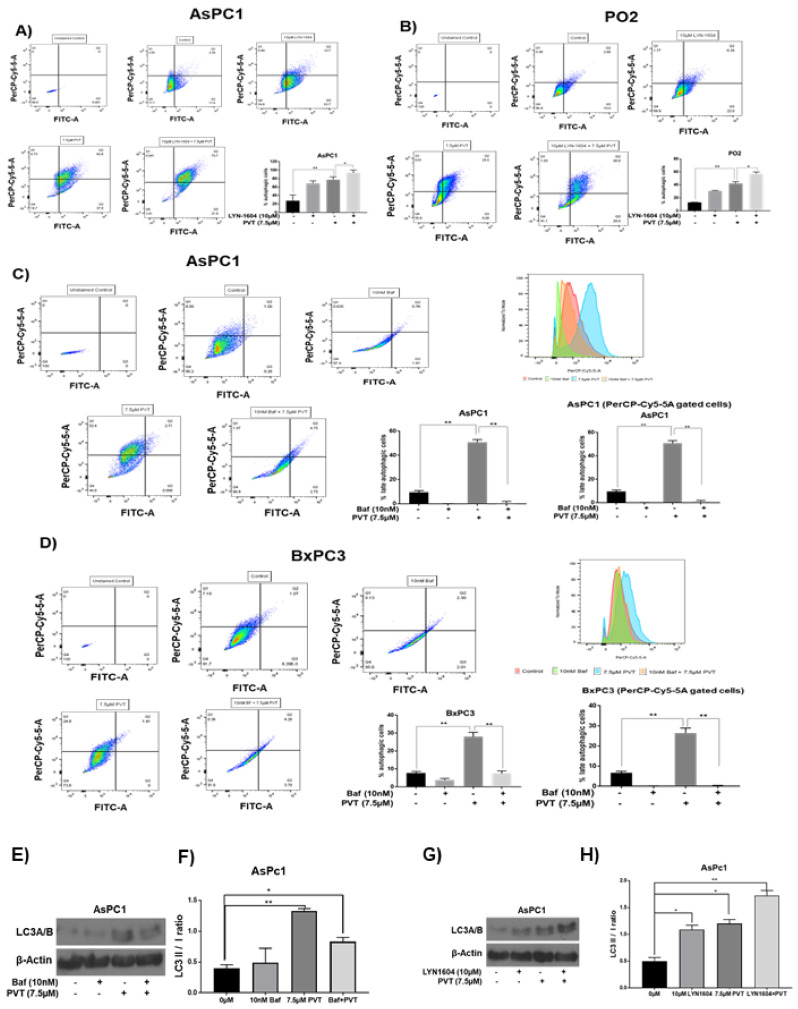
ULK1 agonist LYN-1604 potentiates autophagic effects of PVT in (**A**) AsPC1 and (**B**) PO2 PDAC cells pretreated with 10 µM LYN-1604 for 3 h and then treated with 7.5 µM PVT for 24 h. Blocking autophagy using Bafilomycin suppressed autophagy inducing effects of PVT in (**C**) AsPC1 (**D**) BxPC3 PDAC cells pretreated with 10 nM bafilomycin (Baf) for 3 h and then treated with 7.5 µM PVT for 24 h. Single cell suspension was then analyzed by acridine orange assay using flow-cytometry. (**E**) Western blot analysis of LC3A/B on AsPC1 cells pretreated with 10 nM bafilomycin (Baf) for 3 h and then treated with 7.5 µM PVT for 24 h (**F**) LC3 II/I ratio from Figure 6E. (**G**) Western blot analysis of LC3A/B on AsPC1 cells pretreated with 10 µM LYN-1604 for 3 h and then treated with 7.5 µM PVT for 24 h. (**H**) LC3 II/I ratio from Figure 6G. * *p* ≤ 0.05, ** *p* ≤ 0.01.

To establish the role of ULK1 in PVT-induced autophagy, ULK1 was pharmacologically induced by the ULK1 agonist LYN-1604 in AsPC1 and PO2 cells. A 2.5-fold increase in autophagy was observed in AsPC1 and PO2 cells treated with 10 μM LYN-1604. On the other hand, 7.5 μM PVT treatment resulted in 2.8- and 3.0-fold increase in autophagy in AsPC1 and PO2 cells, respectively. The autophagy-inducing effects of PVT was increased by 3.4- and 4.3-fold in AsPC1 and PO2 cells respectively when these cells were pretreated with LYN-1604 (Figure 6A,B).

### 3.5. Autophagy Inhibition by Bafilomycin Abrogates the Autophagic Effects of PVT

We pharmacologically inhibited autophagy using bafilomycin and evaluated the effects of PVT in modulating autophagy in AsPC1 and BxPC3 cells. Bafilomycin suppresses autophagy by inhibiting lysosomal acidification. As shown in Figure 6C,D, blocking autophagy using 10 nM bafilomycin abrogated the autophagic effects of PVT by 3.8–9.0-fold. Interestingly, 10 nM bafilomycin completely inhibited PVT-mediated 3.5–5.4-fold increase in acidified lysosomes, when PerCP-Cy5-5A cells exhibiting red fluorescence (acidified lysosomes) were gated (Figure 6C,D). The autophagy induction in AsPC1 cells was also confirmed by LC3II to LC3I conversion in the presence of bafilomycin or LYN-1604. As shown in Figure 6E,F, treatment of AsPC1 cells with bafilomycin alone did not increase autophagy, however PVT has significantly increased the autophagy induction as shown by increase in LC3I to LC3II conversion. These effects were reduced when PVT was combined with bafilomycin. On the other hand, LYN-1604 an inducer of autophagy initiator gene ULK1, increased the LC3I to LC3II conversion which was further enhanced when AsPC1 cells were treated with both LYN1604 and PVT (Figure 6G,H). These results confirm the autophagic effects of PVT.

### 3.6. PVT Inhibits Cells Proliferation and Induces Apoptotic Mode of Cell Death in PDAC Cells

**Figure 7:**
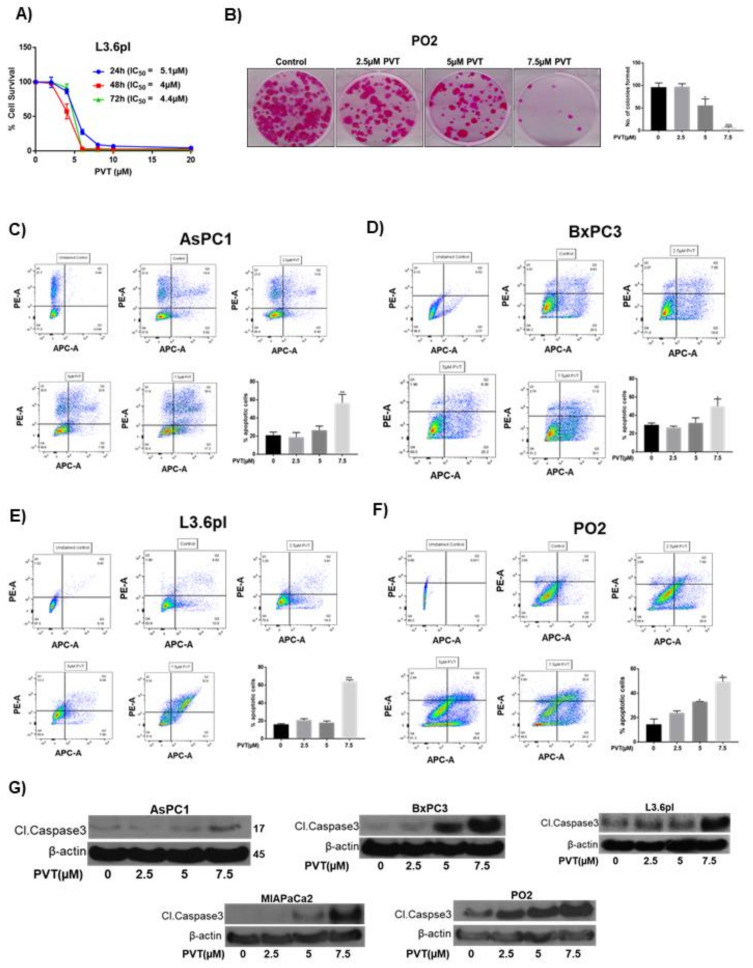
Anti-proliferative and apoptotic effects of PVT in PDAC cells (**A**) Cytotoxic effects of PVT were evaluated in L3.6pl cells by SRB assay. (**B**) Colony formation assay on PO2 cells. (**C**) AsPC1 (**D**) BxPC3 (**E**) L3.6pl and (**F**) PO2 cells were evaluated by AnnexinV/APC assay. Early- and late-stage apoptosis was added and plotted in graphs. Data were quantitated using FlowJo software and plotted using GraphPad Prism 7.0. Whole cell lysates of (**G**) AsPC1, BxPC3, L3.6pl, MIAPaCa2, and PO2 cells treated with increasing concentrations of PVT were immunoblotted for cleaved caspase 3. Statistically significant when compared with control. * *p* ≤ 0.05, ** *p* ≤ 0.01, *** *p* ≤ 0.001.

Survival of L36.pl cells was significantly reduced upon treatment with increasing concentrations of PVT for 24, 48, and 72 h. The IC_50_ of PVT was identified to be 5.1 µM, 4 µM, and 4.4 µM at 24, 28, and 72 h respectively (Figure 7A). In another experiment, the effect of PVT on colony formation was evaluated by colony formation assay. Our results showed that, 5 µM PVT suppressed 50% of colony formation in PO2 cells. Whereas. 7.5 µM PVT completely diminished the colony forming ability of PDAC cells. These results demonstrate the cytotoxic and anticlonogenic effects of PVT. In our previously published study, we have evaluated the cytotoxic effects of PVT in BxPC3, AsPC1, MiaPaCa2 and PO2 cell lines with the IC_50_ in same range as observed in L36.pl [13]. The cell death caused by PVT was determined by performing AnnexinV/APC assay in AsPC1, BxPC3, L3.6pl and PO2 cells using flow cytometry. Our results showed that PVT induced 1.7–4.0-fold apoptosis in all pancreatic cancer cell lines tested (Figure 7C–F). Treatment of AsPC1 and BxPC3 cells with 7.5 μM PVT resulted in 1.7–2.7-fold increase in apoptosis, whereas an increase of 3.3–4.0 fold was observed in L3.6pl and PO2 cells (Figure 7C–F). Similarly, 5 μM PVT increased the proportion of apoptotic cells by 2.3-fold in PO2 cells (Figure 7F). Apoptotic effects of PVT were also confirmed by an increase in cleavage of caspase 3 in all PDAC cells (Figure 7G).

### 3.7. Modulating Autophagy Abrogates or Enhances the Apoptotic Effects of PVT

**Figure 8:**
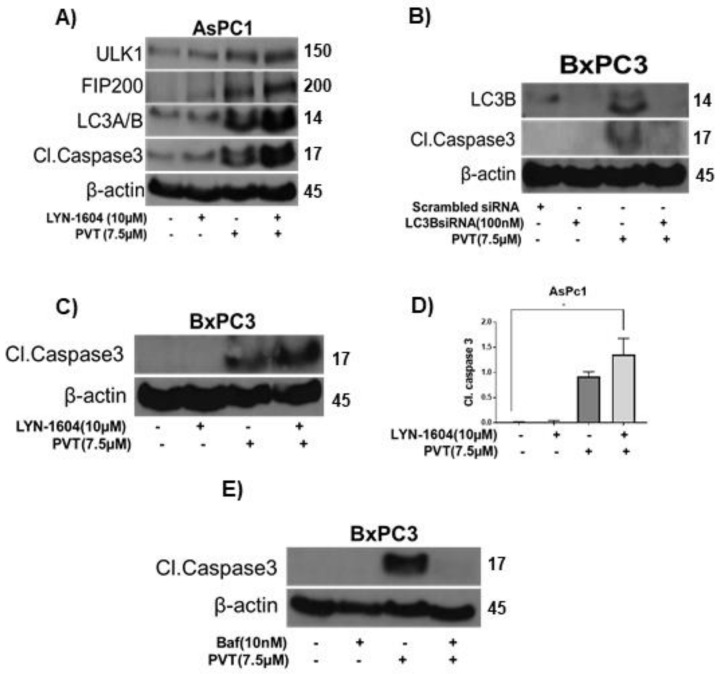
Modulating autophagy enhances or inhibits the apoptotic effects of PVT in BxPC3 cells. Western blot analysis on AsPC1 or BxPC3 whole cell lysates pretreated with (**A**,**C**) 10 µM LYN-1604 (**B**) LC3B siRNA (**E**) 10 nM bafilomycin (Baf) for 3 h and then treated with 7.5 µM PVT for 24 h. (**D**) Quantitated blots from Figure 8C. * *p* ≤ 0.05.

Next, we wanted to prove the role of autophagy in PVT-mediated apoptosis. Our results indicated that LYN-1604, an inducer of autophagy, potentiated the effects of PVT in inducing autophagy and apoptosis, as indicated by the enhanced expression of autophagy markers ULK1, FIP200, and LC3A/B, as compared to that of PVT treatment alone in AsPC1 cells (Figure 8A). Moreover, LYN-1604 enhanced the cleavage of caspase 3 in AsPC1 and BxPC3 cells treated with PVT (Figure 8A,C). In another experiment, we silenced LC3B using LC3B siRNA or pharmacologically inhibited autophagy by bafilomycin followed by treatment with 7.5 μM PVT for 24 h. LC3B silencing was confirmed with reduced expression of LC3B (Figure 8B). As expected, PVT treatment alone increased the expression of LC3B and cleavage of caspase 3. Interestingly, LC3B silencing blocked the apoptosis inducing effects of PVT as indicated by complete inhibition of cleavage of caspase 3 in BxPC3 cells (Figure 8B). Similarly, blocking autophagy with 10 nM bafilomycin abrogated the apoptotic effects of PVT, as depicted by complete inhibition of cleavage of caspase 3 in BxPC3 cells (Figure 8E). Taken together, these results indicate that PVT treatment induced autophagy-mediated apoptosis in PDAC cells.

### 3.8. PVT Suppresses the Growth of BxPC3 Pancreatic Tumors by Inducing Autophagy-Mediated Apoptosis

**Figure 9:**
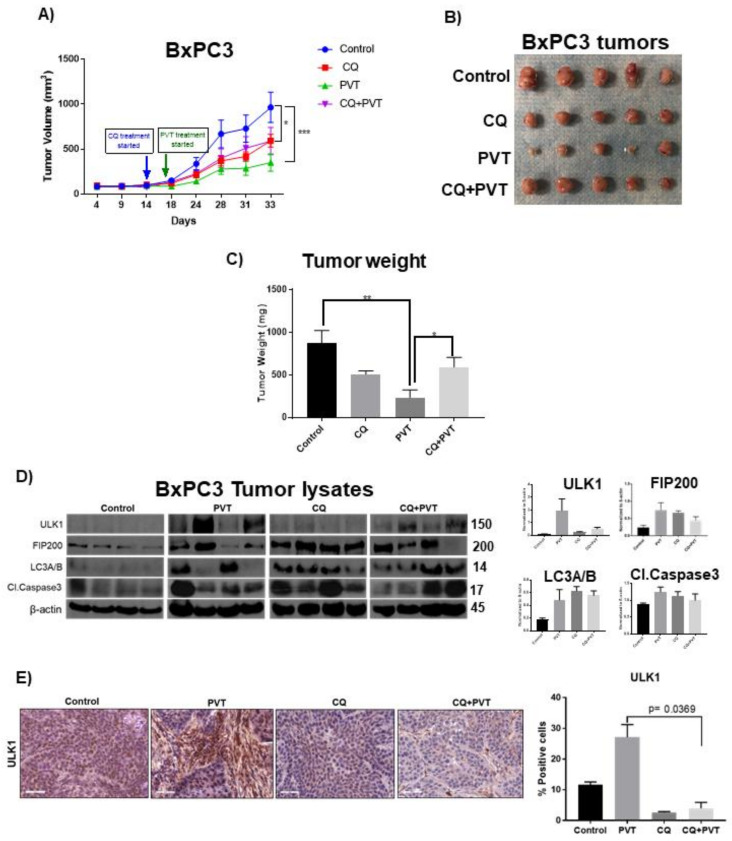
PVT suppresses growth of BxPC3 tumor xenografts by autophagy-mediated apoptosis. (**A**) Growth suppressive effects of PVT in subcutaneous PDAC xenograft model (Control, CQ, PVT, CQ + PVT (*n* = 6)). (**B**) Representative tumor images. (**C**) Tumors were excised and the weight was compared between control, chloroquine (CQ), PVT, and chloroquine + PVT (CQ + PVT) groups. (**D**) Western blot analysis of ULK1, FIP200, LC3A/B and cleaved caspase 3 in BxPC3 tumor lysates. β-actin was used as a loading control. Protein expression in control and treatment groups were normalized with its respective β-actin. Each band represents tumor from individual mouse. (**E**) ULK1 expression determined by IHC in excised tumors. Statistically significant. *p* ≤ 0.05. * *p* ≤ 0.05, ** *p* ≤ 0.01, *** *p* ≤ 0.001.

To evaluate the efficacy of PVT in suppressing pancreatic tumor growth and confirm autophagy-mediated apoptosis in vivo, we implanted BxPC3 cells subcutaneously in athymic nude mice. Our in vivo results demonstrated that, oral administration of PVT treatment suppressed the growth of BxPC3 tumors by 64% when compared to that of control. Interestingly, chloroquine, an inhibitor of autophagy, when given prior to PVT treatment, rescued PVT mediated growth suppression of BxPC3 tumors by 25% (Figure 9A). PVT treatment showed a 73% reduction in tumor weight, whereas chloroquine + PVT treatment group showed a reduction of only 32% when compared to that of control (Figure 9C). The tumors from control, PVT, chloroquine and chloroquine + PVT treated mice were analyzed. Our western blotting results showed that chloroquine reduced the expression of ULK1 and FIP200 induced by PVT treatment (Figure 9D,E). Moreover, chloroquine blocked the apoptosis induced by PVT as indicated by a decrease in the cleavage of caspase 3 (Figure 9D). These results suggest that PVT treatment suppressed the growth of pancreatic tumors by inducing autophagy-mediated apoptosis.

## 4. Discussion

PDAC is one of the most aggressive forms of cancer with a survival rate of only 6%. The low survival rate is attributed to diagnosis of the cancer at advanced stages, and therapy resistance [22,23]. The complex tumor microenvironment of PDAC is one of the major factors for poor outcome of PDAC management. It is characterized by desmoplasia, which includes poor vasculature, dense fibrotic stroma, nutrient deprivation and harsh hypoxic microenvironment [24]. PDAC cells utilize autophagy as a means to counteract hypoxic, acidic and nutrient-deprived tumor environment.

Under normal conditions, autophagy serves as a checkpoint mechanism to maintain the housekeeping of the cells. Given the catabolic nature of autophagy and the high rate of metabolic functions in pancreas, autophagy regulates various physiological functions of pancreas to meet its high-energy demand. Autophagy plays an integral role in maintaining the pool of pancreatic β cells, which is responsible for insulin synthesis. Loss of autophagic activity was associated with depletion of pancreatic β cells resulting in insulin deficiency, eventually leading to Diabetes mellitus [25]. Meanwhile, impaired autophagic activity caused by blockage of ATG proteins was reported in damaged pancreas as characterized by acinar cell degeneration, fibrosis, pancreatic atrophy, and inflammation [26]. However, in conditions like cancer, autophagy acts as a double-edged sword.

Several studies showed autophagy as a cell survival mechanism in various tumor types [27,28,29,30]. In the event of nutrient deprivation due to poor vascularization, a widely observed phenomenon in tumors, autophagy fuels the cancer cells with energy required for survival [31]. The genetic reprogramming in cancer cells utilizes autophagy to invade and metastasize to other parts of the body [32]. Thereby, autophagy inhibition was demonstrated as a strategy to suppress tumor growth. On the other hand, reduced autophagic activity was reported in hepatocellular carcinoma cells [33]. Cancer Genome Atlas and Tissue Microarray analysis data shows that autophagy initiator protein, ULK1 was downregulated in breast cancer tissues [34]. Cytotoxic autophagy mediated by induction of ULK1 was shown in several cancer cells including PDAC [35,36,37]. This process of cytotoxic autophagy is referred to as Autophagic Cell Death (ACD), a cell death modality independent of apoptosis and necrosis. However, apart from ACD, autophagy also activates apoptosis in cancer cells by different mechanisms [8,10,38,39]. Interestingly, several studies demonstrated the role of ULK1 in caspase and PARP-mediated apoptosis. Particularly, ULK1 agonist LYN-1604 was shown to interact with caspase 3 in breast cancer. In addition, fluoxetine, a selective serotonin reuptake inhibitor was shown to inhibit breast cancer growth by inducing ULK1/autophagy-mediated apoptosis [40,41].

PDAC possesses high basal autophagy compared to that of other epithelial cancers. A corollary to this observation is that peripheral pancreatic tumor tissues exhibit strong expression of LC3 as compared to normal pancreatic cells or other tumor cells [42]. Moreover, cancer cells often encompass defective autophagic capacities, where inducing further autophagy was shown to enhance cytotoxic effects and is referred to as cytotoxic autophagy [43]. Several autophagy inducers were studied for their anticancer effects in pre-clinical studies. The inducers of autophagy include rapamycin, metformin, obatoclax, liensinine, isoliensinine, dauricine, and cepharanthine. These inducers initiate autophagy and mediate cell death by inhibiting oncogenic signaling pathways such as PI3K/Akt/mTOR, JNK, AMPK, Src/CEBPD, STAT3, and increasing the accumulation of autophagy inducing proteins such as Atg5, LC3II, and Beclin-1 [12,44].

Autophagy was linked with several neurodegenerative diseases such as dementia, schizophrenia, chronic psychosis, and bipolar disorder. One of the molecular mechanisms behind the progression of these diseases is reduced expression of autophagy markers such as Beclin-1, ULK-1, and LC3 [45]. Antipsychotic agents like fluspirilene, trifluoperazine, and pimozide are known inducers of autophagy [12,46]. Patients taking antipsychotic medications exhibit reduced psychotic symptoms along with increased autophagy as depicted by the enhanced expression of autophagy markers such as ULK-1, Atg proteins, LC3B, and Beclin-1. Notably, in our previously published study, we demonstrated that penfluridol, an antipsychotic drug, suppressed pancreatic tumor growth by inducing autophagy mediated apoptosis [10]. In the current study, we observed that PVT suppressed the growth of pancreatic tumors by inducing autophagy—mediated apoptosis. Accordingly, the proliferation of AsPC1, BxPC3, L3.6pl, MIAPaCa2, PANC1, and PO2 PDAC cells was suppressed by PVT treatment.

Autophagy is initiated by the activation of ULK1 complex. ULK, a Serine/Threonine kinase is a mammalian homolog of yeast Atg-1. Upon stimulation for autophagy induction, ULK1 interacts with the FAK family kinase interacting protein of 200 kDa (FIP200) and Atg 101 to initiate autophagy. Our results showed that PVT significantly increased the expression of ULK1, FIP200 and Atg 101 in all PDAC cells. Once formed, ULK1 complex then activates class-III PI3K complex, scaffolded by a tumor suppressor protein Beclin-1. Our results do show increased expression of Beclin-1 with PVT treatment. In a ubiquitin like conjugation system, Atg5-Atg12-Atg16 complex lipidate LC3, facilitating the formation of autophagosomes. Autophagosomes then engulf the damaged cellular organelles and protein aggregates, and fuses with the acidified lysosomes to form autophagolysosomes. Sequestered organelles are delivered by the autophagosomes to lysosomes for degradation. Interestingly, PVT treatment in our experiments showed increased expression of Atg5, LC3A/B, the number of autophagosomes and the fusion of autophagosomes with lysosomes. Lysosomal acidification is a crucial intracellular process in autophagy, where the acidic hydrolases degrade the damaged organelles. Acridine Orange (AO) is a lysosomotropic dye, where it becomes concentrated and emits red fluorescence in AVOs like autolysosomes. Our results showed that PVT substantially increased the fluorescence of AO, as evaluated by flow cytometry and confocal microscopy. The increased expression of ULK1 and LC3B with PVT treatment was validated by immunofluorescence. To prove ULK1 as a target of PVT, autophagic and apoptotic effects of PVT were examined in presence of ULK1 agonist LYN-1604. Our results indicated that, LYN-1604 potentiated the autophagic effects of PVT, as demonstrated by enhanced AO staining. Moreover, LYN-1604 pre-treatment augmented the apoptosis inducing effects of PVT, as indicated by increased cleavage of caspase 3 in PDAC cells. Furthermore, blocking autophagy using lysosomal acidification inhibitors, bafilomycin reduced the lysosomal acidification induced by PVT. In addition, LC3B silencing or bafilomycin treatment abrogated the apoptotic effects of PVT, as exhibited by a complete inhibition in the cleavage of caspase-3. These findings indicate that the apoptotic effects of PVT were mediated by ULK1 regulated autophagy. PDAC xenograft model in nude mice was used to evaluate the preclinical efficacy of PVT in vivo. Oral administration of PVT suppressed the growth of BxPC3 tumors by 64%. Interestingly, chloroquine also suppressed 40% growth of pancreatic tumors. These results are similar to that observed by Balic et al., showing that chloroquine alone retards the growth of pancreatic tumors by inhibiting pancreatic cancer stem cells, however, independent of autophagy [47]. So, it is likely that the tumor growth suppressive effects of chloroquine that we observed in our study may be independent of autophagy. Nonetheless, our observations indicated that chloroquine pretreatment blocked the tumor growth suppressive effects of PVT by 25%. Chloroquine reduced the enhanced expression of ULK1, FIP200 and cleaved caspase-3 in the tumors of mice treated with PVT. Surprisingly, chloroquine treatment alone slightly increased the cleavage of caspase 3, which can explain the reduced effect of chloroquine in abrogating the apoptotic effects of PVT. In our study, apoptotic effects of chloroquine can be attributed to the chronic administration of chloroquine in our experiment. Additionally, several studies have established that the apoptotic and autophagic effects of chloroquine are two independent mechanisms which cannot be correlated [48].

Several studies tried to delineate the role of autophagy in inducing apoptosis. The Nomenclature Committee on Cell Death has recommended that the term autophagic cell death should be used only if cell death occurs independent of other cell death pathways like apoptosis and modulating autophagy should eventually affect cell death [49]. Consistent with these recommendations, several studies demonstrated that autophagic cell death is independent of other cell death mechanisms, and cells undergoing autophagic cell death exhibit different morphological and biochemical characteristics [50,51,52,53]. However, some studies suggested that the regulation of cell death pathways like autophagy, apoptosis, ferroptosis and necrosis may be interconnected in determining the fate of the cells [53]. Several other studies demonstrated the induction of autophagy-mediated apoptosis by few drugs in cancer [9,10,12,38]. Gemcitabine, which is currently prescribed as the first-line treatment for pancreatic cancer was shown to induce VMP-1 regulated autophagy mediated apoptosis in human PDAC cells [9]. Although gemcitabine was a relatively effective chemotherapeutic agent, development of resistance to gemcitabine by PDAC patients makes the treatment ineffective. Based on our results, it is likely that PVT may sensitize PDAC cells to gemcitabine therapy. Nonetheless, more studies are warranted. In another study, it was shown that MonoD, a novel analog of digitoxin suppressed the growth of lung cancer cells by inducing autophagy mediated apoptosis. In addition, these and other studies evidently proved that autophagy inhibitors can suppress the apoptosis inducing effects of several drugs [9,10,12,38].

In agreement with these studies, our results provide convincing evidence that PVT suppresses the growth of PDAC in vitro and in vivo by inducing autophagy-mediated apoptosis and could serve as a novel therapeutic option for pancreatic cancer. Our study is the first report to demonstrate the autophagy-mediated antineoplastic effects of PVT in pancreatic cancer.

## 5. Conclusions

In this study, we showed that PVT, an antipsychotic drug, has novel anticancer potential. It mediates its antineoplastic effects by inducing autophagy as demonstrated by immunoblotting, flow cytometry, and immunofluorescence microscopy. Our results indicate that PVT delivers its proautophagic effects by increasing the expression of autophagy related proteins ATG, ULK1, and LC3A/B. Additionally, PVT also increased the lysosomal acidification as shown by increased acridine orange staining. Similar observations were made in our in vivo studies. Taken together, this study shows that PVT can suppress the progression of pancreatic cancer by modulating autophagy.

## Data Availability

The publicly available data can be obtained from http://gepia.cancer-pku.cn/ (GEPIA) (accessed on 14 October 2021) and https://hgserver1.amc.nl/cgi-bin/r2/main.cgi (R2) (accessed on 14 October 2021). The authors agree to make data available upon request.
